# Supplementary material for: Fungal Diversity and Its Relationship with Environmental Factors in Coastal Sediments from Guangdong, China
Source: J Fungi (Basel). 2023 Jan 11;9(1):101. doi: 10.3390/jof9010101 (PMC9866456; doi:10.3390/jof9010101)
Supplement: Supplementary file 1 [file jof-09-00101-s001.zip › jof-2154854-supplementary.pdf]

Supplementary Material

Table S1. The latitudes, longitudes and type of coastal sediments in Guangdong

Sites	Latitudes/ Longitudes	Type of sediments
S1	22°50′13″N/116°7′34″E	Sand
S2	22°40′4″N/115°32′8″E	Sand
S3	22°43′38″N/114°44′31″E	Sand
S4	22°35′17″E/114°16′25″E	Sand
S5	22°23′28″N/113°37′42″E	Sand
S6	22°5′7″N/113°28′49″E	Sand
S7	21°53′8″N/112°53′11″E	Sand
S8	21°44′36″E/112°11′59″	Sand
S9	21°30′53″N/111°27′56″E	Sand
S10	21°23′10″N/110°53′8″E	Sand
S11	21°14′14″N/110°38′11″E	Sand

Table S2. Physicochemical properties of coastal sediments in Guangdong

Site	T (℃)	DO (mg/L)	pH	Salinity (‰)	TOC (mg/kg)	TN (mg/kg)	TP (mg/kg)
S1	22.43±0.15b	8.16±0.07a	8.35±0.07bc	32.75±0.11b	595.52±43.91e	124.11±23.04c	191.87±28.16c
S2	23.23±0.32b	6.34±0.15d	8.38±0.01b	33.12±0.10a	760.65±49.32e	291.97±49.89ab	939.84±197.14ab
S3	24.27±0.15ab	6.81±0.12bc	8.37±0.04bc	32.46±0.43b	1050.15±150.01de	234.44±55.84b	1330.08±634.77a
S4	23.43±0.21ab	5.33±0.14ef	8.18±0.01d	32.77±0.12b	1067.36±111.05d	217.96±43.70b	630.89±317.39b
S5	21.43±0.15c	6.62±0.05d	8.74±0.03a	14.65±0.22e	3132.60±420.64a	313.91±40.51a	907.32±84.49ab
S6	22.43±0.25b	4.90±0.06f	8.08±0.01e	4.56±0.27g	1797.77±172.78b	290.33±45.48ab	1069.92±101.54ab
S7	23.00±0.10b	6.03±0.07de	8.10±0.04de	27.43±0.28d	1260.41±171.45cd	184.59±21.01bc	533.33±156.81bc
S8	24.33±0.15a	6.93±0.06bc	8.26±0.01c	30.72±0.40c	893.08±67.61de	259.69±14.58ab	760.97±258.13b
S9	23.43±0.15ab	6.62±0.04d	8.34±0.02bc	30.32±0.08c	1359.63±108.67cd	293.73±36.43ab	468.29±223.54bc
S10	23.20±0.10b	6.81±0.03c	8.33±0.03bc	25.18±0.82d	975.96±182.72de	191.84±33.64b	354.47±122.76bc
S11	22.80±0.10b	6.98±0.04b	8.03±0.02e	10.50±0.16f	1424.45±166.91c	209.43±18.38b	354.47±28.16bc

The different lowercase letters indicate that the difference is significant at the 0.05 level. n=3. T: Temperature, DO: Dissolved oxygen, TOC: Total organic carbon, TN: Total nitrogen, TP: Total phosphorus.

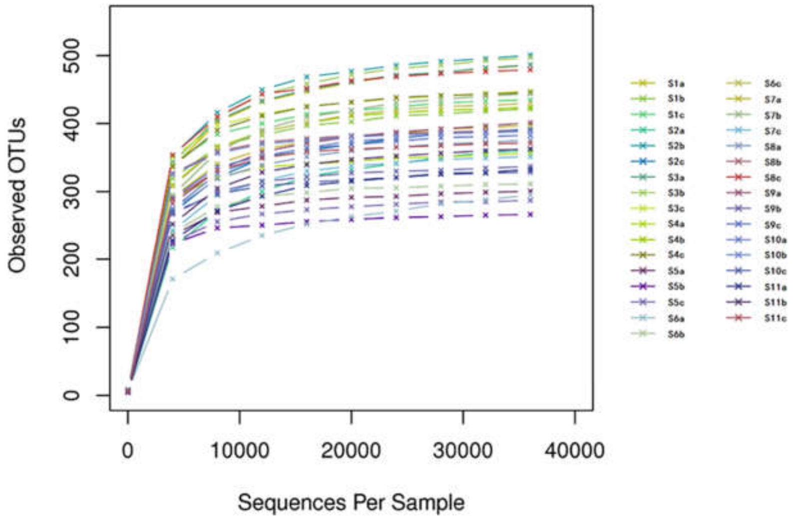

Figure S1. Rarefaction curves of fungi in coastal sediments along the coast of Guangdong

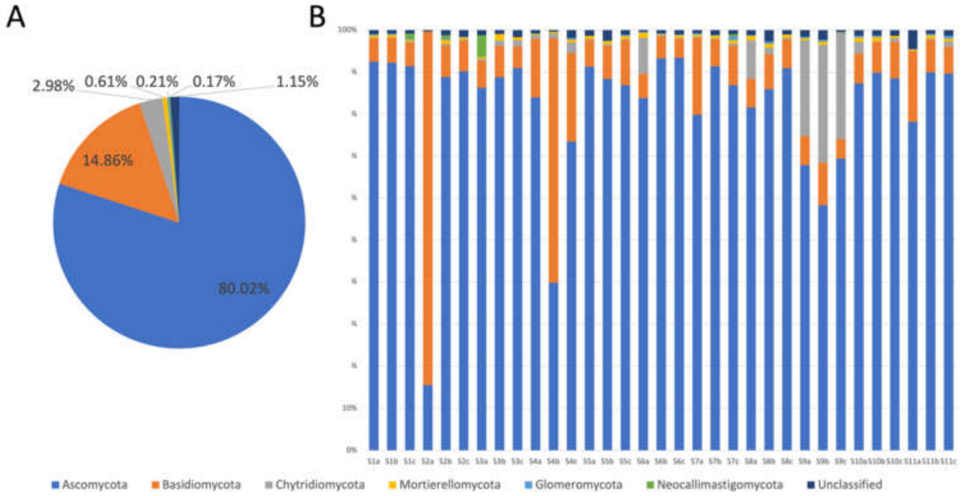

Figure S2. Fungal community composition (A) and distribution (B) at phylum level in 33 coastal sediment samples from 11 different sites (S1-S11) from Guangdong, South China

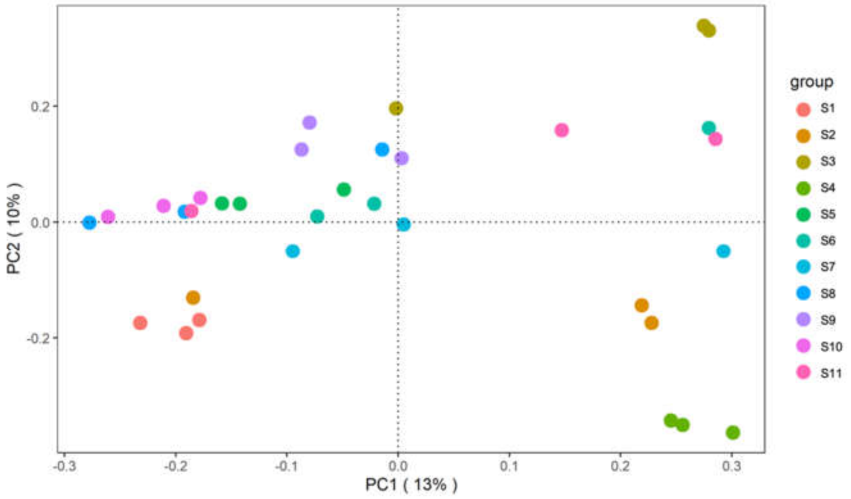

Figure S3. PCoA analysis of fungal communities in 11 coastal sediments (S1-S11) of Guangdong
